# Anomalous Structural Transition and Electrical Transport Behaviors in Compressed Zn_2_SnO_4_: Effect of Interface

**DOI:** 10.1038/srep14417

**Published:** 2015-09-24

**Authors:** Haiwa Zhang, Feng Ke, Yan Li, Li Wang, Cailong Liu, Yi Zeng, Mingguang Yao, Yonghao Han, Yanzhang Ma, Chunxiao Gao

**Affiliations:** 1 State Key Lab for Superhard Materials, Institute of Atomic and Molecular Physics and Department of Materials Science, Jilin University, Changchun 130012, China; 2 Center for High Pressure Science and Technology Advanced Research, Shanghai 201203, China; 3 Department of Mechanical Engineering, Texas Tech University, Lubbock, TX 79409, USA

## Abstract

The interface effect is one of the most important factors that strongly affect the structural transformations and the properties of nano-/submicro-crystals under pressure. However, characterization of the granular boundary changes in materials is always challenging. Here, using tetrakaidecahedral Zn_2_SnO_4_ microcrystals as an example, we employed alternating current impedance, X-ray diffraction methods and transmission electron microscopy to elucidate the effect of the interface on the structure and electrical transport behavior of the Zn_2_SnO_4_ material under pressure. We clearly show that grain refinement of the initial microcrystals into nanocrystals (approximately 5 *n*m) occurs at above 12.5 GPa and is characterized by an anomalous resistance variation without a structural phase transition. A new phase transition pathway from the cubic to hexagonal structure occurs at approximately 29.8 GPa in Zn_2_SnO_4_. The unexpected grain refinement may explain the new structural transition in Zn_2_SnO_4_, which is different from the previous theoretical prediction. Our results provide new insights into the link between the structural transition, interface changes and electrical transport properties of Zn_2_SnO_4_.

Understanding phase transformations in nano-/submicro-crystal systems is an area of considerable scientific interest due to the appearance of many novel behaviors at high pressure in these systems. In particular, the phase transition pressure and processing of nano-/submicro-materials strongly depend on their grain size, shape, and structure^1^,^2^,^3^,^4^. This has led to intensive study of the effects of grain size and shape on the critical phase transition pressures, phase transition mechanisms, and amorphization processes. For example, crystal size and morphology have been suggested to affect the structural transitions of materials such as ZnO^5^, Y_2_O_3_^6^ and graphite^7^ upon compression. In fact, the size and shape effects are closely correlated with the exposed face and granular interface of nano-/submicro-materials; however, these have rarely been explored in the transformation of nanomaterials due to the great difficulties in detecting and characterizing the related changes under pressure. Although changes in the granular boundary and interface may strongly affect the phase transition pathway and the properties of materials, they have not been well understood.

Zn_2_SnO_4_ (ZTO) is an important n-type transparent conducting oxide, which has been attracting intense interest due to its outstanding performance and potential use in applications such as optoelectronic devices, anode materials for dye-sensitized solar cells, and photocatalysts for the degradation of organic pollutants^8^,^9^,^10^. At ambient conditions, ZTO crystallizes into a typical inverse spinel structure with the *Fd*3*m* space group^11^,^12^,^13^. Recent theoretical and experimental studies suggest that ZTO undergoes several structural transitions upon compression. High pressure studies on ZTO nanowires showed that cubic ZTO transformed into an intermediate orthorhombic phase at 12.9 GPa and then to a new high-pressure orthorhombic structure at approximately 32.7 GPa. The structure of the high-pressure phase is similar to the ambient-pressure CaFe_2_O_4_ (ferrite-type) structure^14^. In sharp contrast, theoretical simulations show that at above 39.0 GPa, ZTO transforms into another new orthorhombic structure that is similar to that of titanite-type CaTi_2_O_4_^15^. Thus, further studies are required to elucidate the underlying mechanisms in order to understand the difference in the reported phase transitions. Moreover, the grain boundary or interface effects should play important roles in the structural transitions and property variations of ZTO materials. However, these effects have not been studied until now. Alternating current (AC) impedance spectroscopy is an effective technique for the detection of the changes at the grain boundary or the interface upon compression and can be used to study the related property changes by measuring the boundary transport behaviors such as the boundary resistance.

In this work, we used high pressure AC impedance and X-ray diffraction measurements combined with high-resolution transmission electron microscopy (HRTEM) observations to investigate tetrakaidecahedral ZTO single crystals. We studied the effect of the crystal grain boundary on the structure and electrical transport properties of the ZTO crystals upon compression. A new phase transition pathway was found in ZTO crystals under pressure, and the effect of the interface on the transformation was elucidated.

## Experimental Results and Discussion

**Figure 1:**
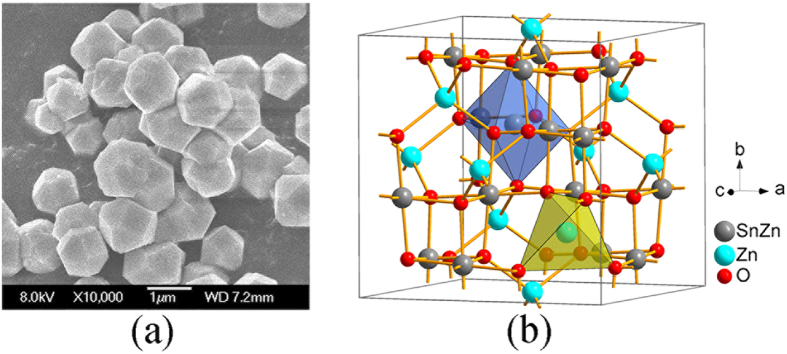
Morphology and crystal structure of ZTO. (**a**) TEM image of ZTO single crystals. (**b**) Simulated inverse spinel structure of ZTO at ambient pressure.

The tetrakaidecahedral ZTO single crystals were synthesized by a hydrothermal method^16^. The obtained sample exhibits a cubic structure, in good agreement with previous work (JCPDS 24–1470). As shown in Fig. 1a, the average size of ZTO single crystals is approximately 800 *n*m. Figure 1b shows the simulated crystal structure of ZTO with an inverse spinel structure consisting of alternating tetrahedral ZnO_4_ and octahedral ZnO_6_ or SnO_6_ sublattices at ambient pressure.

## *In Situ* High-Pressure XRD Measurements

**Figure 2:**
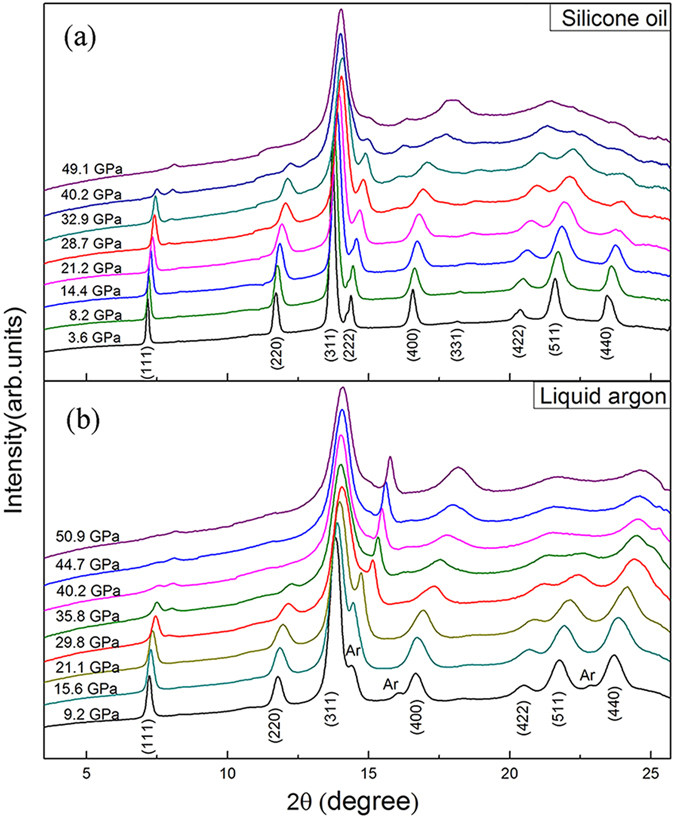
Representative XRD patterns of the tetrakaidecahedral ZTO single crystals upon compression with different PTMs. (**a**) Silicone oil as the PTM up to 49.1 GPa. (**b**) Liquid argon as the PTM up to 50.9 GPa.

**Figure 3:**
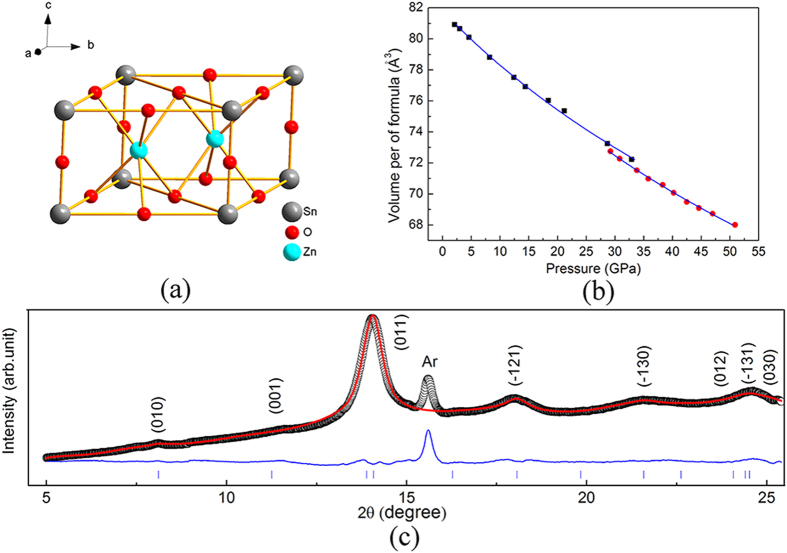
Data for ZTO under high pressure. (**a**) High-pressure hexagonal structure of ZTO. (**b**) ZTO crystal volume curves as functions of pressure. Solid lines are the results fitted with a second-order Birch-Murnaghan equation. (**c**) Rietveld refinement results of the high-pressure phase at 44.7 GPa. Solid lines and open circles represent the fitted and experimental data, respectively. Solid lines at the bottom are the residual intensities. Vertical bars indicate the peak positions.

For direct comparison with previous studies, we first conducted high-pressure XRD experiments on tetrakaidecahedral ZTO single crystals for pressures up to 49.1 GPa with silicone oil as the pressure-transmitting medium (PTM). Figure 2a shows the selected XRD patterns of ZTO upon compression. Using the Rietveld refinement analysis implemented in the GSAS program, the diffraction pattern obtained at ambient pressure was found to be well-indexed by a cubic structure with the *Fd*3*m* space group. As pressure increases, all diffraction peaks shift toward smaller d-spacings, and at approximately 28.7 GPa, two new diffraction peaks appear, indicating a structural transition. The high-pressure phase is stable at pressures up to 49.1 GPa, the highest pressure used in this experiment. To confirm the results, liquid argon was also used as the PTM in the high pressure experiments. The recorded XRD patterns are displayed in Fig. 2b. It is clear that the XRD pattern agrees well with that obtained when using silicone oil as the PTM, the phase transition occurs at approximately 29.8 GPa. Furthermore, our Rietveld refinement analysis of the high pressure pattern suggests that all the peaks of the new phase can be indexed by a hexagonal structure (Fig. 3c). The best fit to the high pressure phase gives a hexagonal structure with the *P6/mmm* space group. The simulated structure of ZTO in such a hexagonal phase is shown in Fig. 3a. Additionally, examination of the pressure-volume curves presented in Fig. 3b shows that the cubic-hexagonal structural transition results in only a small volume reduction. Moreover, we noticed that in other materials such as rare-earth oxides, pressure-induced cubic-hexagonal structural transitions have also been frequently observed upon compression^17^,^18^. Such a transition has been suggested to undergo a reconstructive process. The occurrence of the transition can be understood as being caused by the smaller molar volume of the hexagonal phase compared with the cubic phase and a relatively small energy difference between the hexagonal phase and the cubic phase^18^.

The structural transition of our ZTO sample is clearly different from the structural transition in ZTO nanowires reported by Shen *et al.* in ref. 14, in which transformations into an intermediate phase at 12.9 GPa and then into an orthorhombic structure at above 32.7 GPa were observed. Intuitively, this difference in the phase transitions can be attributed to the different crystal sizes and morphologies of the ZTO samples used in the two experiments. In our case, the ZTO single crystals have a tetrakaidecahedral morphology with an average particle size of approximately 800 *n*m, while the nanowires used in ref. 14 have an average diameter of approximately 150 *n*m. It is known that size and morphology effects on the phase transition of a material are probably due to the different arrangements of surface atoms and the ratio of the number of atoms on the surface to that in the bulk of the nano-/submicro-structured crystals, corresponding to the ratio of the surface energy and the cohesive energy^1^,^19^. The cohesive energy is inversely proportional to the surface/volume ratio^20^, and thus, we can estimate that the surface/volume ratio for our ZTO sample is on the same order of magnitude as that for the sample used by Shen *et al.* However, no direct information is available for comparison of the surface energies for the two ZTO samples. Furthermore, although the signal for the presence of a phase transition in the nanowires at 12.9 GPa was somewhat ambiguous^14^, the broadening and weakening of several diffraction peaks indicate that there are some changes in the ZTO samples. We therefore used AC impedance measurements and HRTEM to explore the underlying changes in the ZTO sample.

## AC Impedance Spectroscopy Measurement at High Pressures

As is well known, structural transitions of a material are usually accompanied by changes in the electronic structure that can be reflected in electrical transport measurements. In particular, the transitions of the boundaries or the interface of crystals can modify the boundary transport properties; subsequently, the correlation between the microstructure and the electrical response can be revealed^21^. We thus conducted AC impedance spectroscopy measurements to study the electrical transport behaviors of ZTO under pressure.

**Figure 4:**
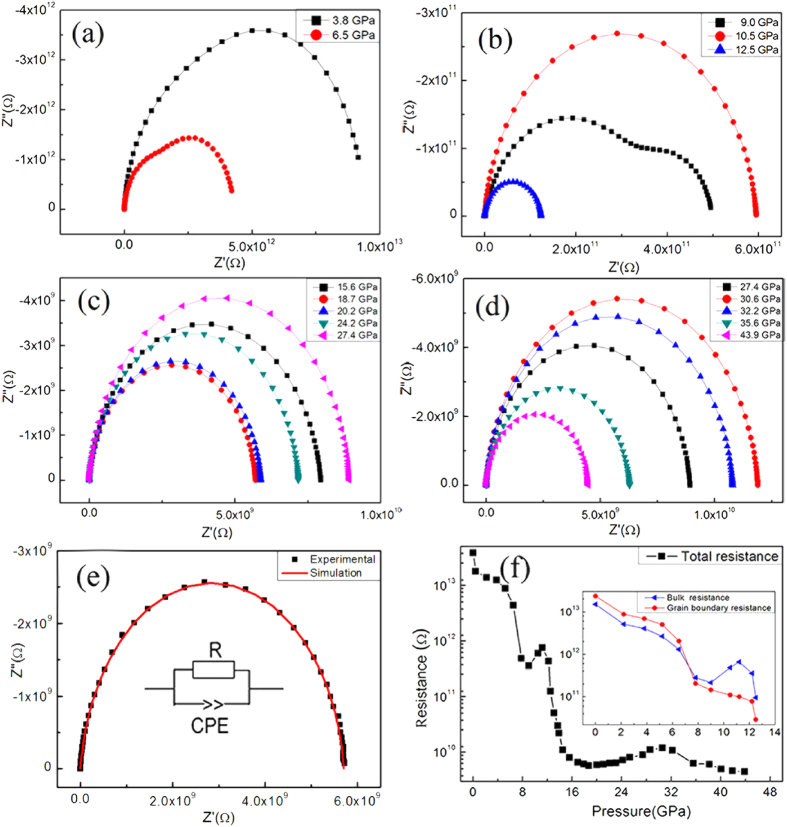
Electrical data of ZTO upon compression. (**a**–**d**) Complex impedance planes of ZTO at different pressures. (**e**) Equivalent circuit and fitting results. (**f**) Total resistance of ZTO under high pressure. Inset shows the bulk and grain boundary resistances of ZTO under pressure.

Figure 4a–d display the typical Nyquist plots of ZTO at different pressures. Two overlapping semicircles can be observed on the complex impedance plane: the arcs in the high frequency area on the left-hand side represent the contribution of the bulk, and the arcs in the low frequency on the right-hand side describe the boundary contribution^22^. As pressure increases up to 9.0 GPa, both the high- and low-frequency arcs contract dramatically. However, the arcs due to the boundary contribution shrink much faster than those due to the bulk. Above 12.5 GPa, the arcs corresponding to the boundary contribution are much weaker compared with those of the bulk contribution, and hence, bulk conduction dominates the electrical transport behavior of ZTO.

To quantify the variations of the boundary and bulk contributions under pressure, an equivalent circuit was used to fit the obtained ZTO impedance spectra. Figure 4e shows that the fitting results agree well with the experimental results. The results obtained for the corresponding resistance are plotted in Fig. 4f. Inspection of Fig. 4f shows distinct changes of the total resistance with increasing pressure. Three obvious changes in the slopes of the pressure dependence of the total resistance can be clearly seen at 9.0, 18.7 and 30.6 GPa. In the 0–18.7 GPa pressure range, the total resistance tends to decrease with increasing pressure. Remarkably, there is a pronounced change in the 9.0–12.5 GPa pressure range, where the total resistance actually increases. To understand this unusual transition, we show the changes of bulk resistance and grain boundary resistance from ambient pressure to 12.5 GPa in the inset of Fig. 4f. While the grain boundary resistance decreases with increasing pressure, the bulk resistance increases from 9.0 to 12.5 GPa, leading to an increase of the total resistance. From 18.7 to 30.6 GPa, the total resistance increases smoothly with increasing pressure, while it clearly decreases at higher pressures (30.6–43.9 GPa).

**Figure 5:**
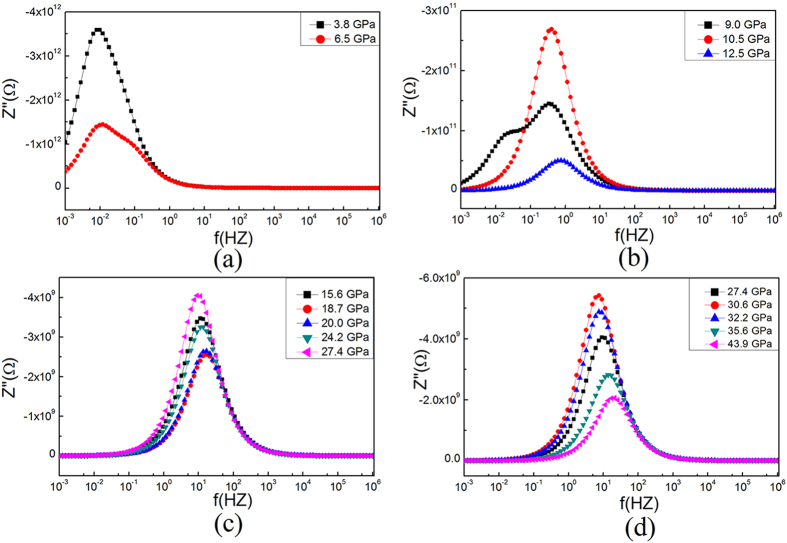
Imaginary part of impedance as a function of relaxation frequency at various pressures .

Such pressure-induced variation in the electrical transport behavior of the compressed ZTO can be further observed using Bode plots (*Z*” vs. *f* curves) of the impedance spectrum (Fig. 5), where the bulk and boundary relaxation peaks are located at high- and low-frequency zones, respectively. Upon compression, the relaxation peaks of both the bulk and the boundary shift toward the higher frequency zone and gradually decrease in intensity. The boundary relaxation peak intensity drops faster than that of the bulk relaxation peak, suggesting a weakening of the boundary effect and fewer charge carriers scattered by the boundary. The relaxation peaks due to the bulk effect increase anomalously from 9.0 to 12.5 GPa. Above 12.5 GPa, the boundary relaxation peaks almost disappear, suggesting that the bulk effect dominates electrical transport in ZTO. The bulk relaxation peaks gradually shift toward the high frequency zone above 12.5 GPa, followed by a reversal and a downshift to lower frequencies and then an increase again above 18.7 GPa. Above 30.6 GPa, the grain relaxation peaks shift to higher frequencies, suggesting that the resistance decreases again. These observations confirm the attenuation of the grain boundary contribution to the total electrical conduction and the slope changes of the bulk resistance in ZTO at 9.0, 18.7 and 30.6 GPa.

We now discuss the electrical transport variations of ZTO under pressure. It is well-known that the electrical conduction of materials is strongly affected by the interface, crystal grains and band gap changes. At the initial stage of compression, van der Waals interactions between the crystal grains increase with increasing pressure and both the interface and the crystal grains make important contributions to the conduction^23^. When the crystal grains are sufficiently close, strong interactions start to occur between the crystal grains and the dangling bonds on the grain surface, and thus, the charge carrier scattering caused by the grain boundary becomes weak, leading to the decreased grain boundary resistance. Additionally, the approach of grain interfaces and the increased number of defects induced by pressure can make the carrier transport easier, subsequently leading to the decrease of the bulk resistance. When the applied pressure reaches 18.7 GPa, the slope of the bulk resistance changes remarkably and gradually increases with increasing pressure. This can be explained by the fact that (1) the grain boundary effect decreases with pressure and disappears at 12.5 GPa, and above this pressure, the bulk resistance basically determines the electrical transport behavior; (2) theoretically, the band gap of ZTO is predicted to gradually increase with increasing pressure^15^, and therefore, the band gap increase has increasingly stronger influence on electrical transport properties, making thermal excitation of electrons from the highest occupied valence band to the lowest unoccupied conduction band more difficult and thus increasing the ZTO resistance with increasing pressure in the 18.7–30.6 GPa range. Above 30.6 GPa, the cubic to hexagonal structural transition occurs; this substantially changes the electronic structure of ZTO and consequently results in the slope change in the curve for the pressure dependence of the bulk resistance.

## HRTEM Experiment after Decompression

It is interesting that the bulk resistance of ZTO increases with increasing pressure from 9.0 to 12.5 GPa, while XRD measurements indicate that no structural transition occurs from ambient pressure to 29.8 GPa. These anomalous phenomena may be caused by the grain refinement of the samples because changes in the grain boundary or the interface can modify the bulk resistance and the grain boundary resistance and thus the total resistance. To prove this hypothesis, HRTEM observations on ZTO samples decompressed from various pressures have been carried out to study the grain boundary changes.

**Figure 6:**
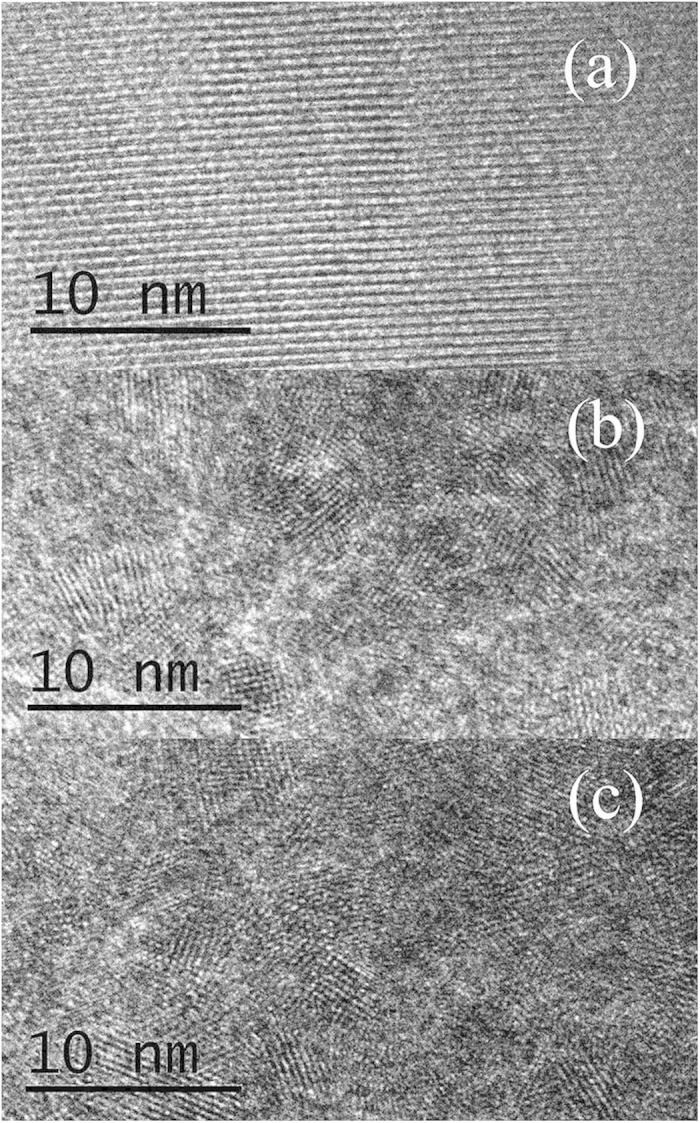
TEM images of the ZTO samples after decompression from different pressures. (**a**) 8.0 GPa; (**b**) 13.0 GPa; (**c**) 25.0 GPa.

As shown in Fig. 6, the samples preserve the single crystal structure with a long range order when decompressed from 8.0 GPa (Fig. 6a) and are found to be crushed into randomly stacked nanocrystals with an average grain size of approximately 5 *n*m when decompressed from 13.0 GPa (Fig. 6b). The nanocrystal sizes are almost constant in the sample when decompressed from pressures higher than 25.0 GPa (Fig. 6c).

Based on HRTEM observations of the decompressed samples, we suggest that nanocrystallization of ZTO samples can significantly increase the number of interfaces in the compressed ZTO, producing a large number of dangling bonds on the grain surface. This enhances the interaction between the dangling bonds, and therefore, the scattering effect on the carriers becomes weak. This indicates that the movement of the carriers in the interface becomes easier, while the transport of carriers through the grains becomes more difficult. This mechanism provides a good explanation for the observed sharp fall in the grain boundary resistance to a level contributing little to the total resistance. In contrast, the contribution of the bulk resistance to the total resistance increases in this pressure range. In the 13.0–25.0 GPa pressure range, the nanocrystals do not undergo any further grain refinement and the electrical transport behaviors are mainly affected by the band gap. This verifies that the gradual increase in the bulk resistance above 18.7 GPa is caused by the widening of the band gap, in agreement with the theoretical calculations^15^. Finally, the grain refinement of the initial microcrystals into nanocrystals with a size of approximately 5 *n*m at above 12.5 GPa may result in significant differences in the surface energy and cohesive energy compared to the bulk counterpart and thus lead to different phase transition pressures and phase transition mechanisms. This may be the underlying reason why the phase transition pathway observed in our experiment is different from those predicted by theoretical calculations.

In summary, AC impedance, X-ray diffraction measurements and HRTEM have been used to reveal the interface effect on the structural transformation and electrical transport behaviors of Zn_2_SnO_4_ microcrystals under pressure. We clearly demonstrated that grain refinement of the initial microcrystals into nanocrystals (approximately 5 *n*m) occurs at above 12.5 GPa and is accompanied by an anomalous resistance variation without a structural transition at this pressure. A new phase transition from the cubic to hexagonal structure occurs in Zn_2_SnO_4_ at approximately 29.8 GPa, coinciding with a significant decrease of the resistance. The unexpected grain refinement may explain the new phase transition pathway observed in Zn_2_SnO_4_ rather than those predicted by theoretical calculations. The results also provide new insights into the link between structural transitions, interface changes and electrical transport properties of Zn_2_SnO_4_. Finally, the hidden granular interface changes may also occur in other materials and could affect the phase transition and properties of the materials under pressure; therefore, our strategy can be extended to uncover such changes and shed light on the underlying mechanism.

## Methods

Diamond anvil cells (DACs) were used to generate high pressure with pre-indented rhenium as the gasket. Diamond anvils of 300 *μ*m flats were used for all the studies. The R1-line emission of a tiny ruby chip (approximately 5 *μ*m) was used for pressure calibration^24^. *In situ* high-pressure XRD experiments were conducted at beamline 4W2 of the Beijing Synchrotron Radiation Facility (BSRF) and beamline BL15U1 of the Shanghai Synchrotron Radiation Facility (SSRF) using an angle-dispersive source (λ = 0.6199 Å). Silicone oil or liquid argon was used as a PTM. FIT2D software was used for integrating Bragg diffraction images, yielding patterns of one-dimensional intensity versus diffraction angle 2θ. XRD patterns were fitted with Rietveld refinement using the GSAS program package.

The AC impedance spectrum was measured with a two-electrode configuration microcircuit, in which the rhenium gasket serves as one electrode and a thin-film electrode integrated onto one diamond anvil surface serves as the other electrode^25^. More details on the fabrication of the film microcircuit and the insulation between the gasket and the film electrode have been previously described^26^,^27^. The AC impedance spectra were measured with a Solartron 1260 impedance analyzer equipped with a Solartron 1296 dielectric interface. An a.c. sine voltage signal with an amplitude of 1 *V* and a range from 0.01 Hz to 10 MHz was applied to the samples.

High-resolution transmission electron microscopy (HRTEM) images were obtained on a JEM-2100F operated at 200 kV. The decompressed sample was first ultrasonically dispersed in acetone and then dropped onto carbon grids.

## Additional Information

**How to cite this article**: Zhang, H. *et al.* Anomalous Structural Transition and Electrical Transport Behaviors in Compressed Zn_2_SnO_4_: Effect of Interface. *Sci. Rep.* **5**, 14417; doi: 10.1038/srep14417 (2015).

## References

[b1] Wang Z. *et al.* Morphology-tuned wurtzite-type ZnS nanobelts. Nature Mater. 4, 922 (2005). 16284620 10.1038/nmat1522

[b2] Wickham J. N., Herhold A. B. & Alivisatos A. P. Shape change as an indicator of mechanism in the high-pressure structural transformations of CdSe nanocrystals. Phys. Rev. Lett. 84, 923 (2000). 11017406 10.1103/PhysRevLett.84.923

[b3] San-Miguel A. Nanomaterials under high-pressure. Chem. Soc. Rev. 35, 876 (2006). 17003894 10.1039/b517779k

[b4] Swamy V., Kuznetsov A. Y., Dubrovinsky L. S., Kurnosov A. & Prakapenka V. B. Unusual compression behavior of anatase TiO_2_ nanocrystals. Phys. Rev. Lett. 103, 075505 (2009). 19792660 10.1103/PhysRevLett.103.075505

[b5] Bayarjargal L., Wiehl L. & Winkler B. Influence of grain Size, surface Energy, and deviatoric stress on the pressure-induced phase transition of ZnO and AlN. High Pressure Res. 33, 642 (2013).

[b6] Wang L. *et al.* Size-dependent amorphization of nanoscale Y_2_O_3_ at high pressure. Phys. Rev. Lett. 105, 095701 (2010). 20868175 10.1103/PhysRevLett.105.095701

[b7] Liu C. L. *et al.* Size-dependent phase transition of graphite to superhard graphite under high pressure at room temperature. J. Appl. Phys. 112, 103707 (2012).

[b8] Zhao Y. *et al.* Band gap tunable Zn_2_SnO_4_ nanocubes through thermal effect and their outstanding ultraviolet light photoresponse. Sci. Rep. 4, 6841 (2014). 25355096 10.1038/srep06847 PMC4213775

[b9] Chen Z., Cao M. H. & Hu C. W. Novel Zn_2_SnO_4_ hierarchical nanostructures and their gas sensing properties toward Ethanol. J. Phys. Chem. C 115, 5522 (2011).

[b10] Alpuche-Aviles M. A. & Wu Y. Y. Photoelectrochemical study of the band structure of Zn_2_SnO_4_ prepared by the hydrothermal method. J. Am. Chem. Soc. 131, 3216 (2009). 19219993 10.1021/ja806719x

[b11] Wei S. H. & Zhang S. B. First-principles study of cation distribution in eighteen closed-shell A^II^B_2_^III^O_4_ and A^IV^B_2_^II^O_4_ spinel oxides. Phys. Rev. B 63, 045112 (2001).

[b12] Zeng J. *et al.* Transformation process and photocatalytic activities of hydrothermally synthesized Zn_2_SnO_4_ nanocrystals. J. Phys. Chem.C 112, 4159 (2008).

[b13] Segev D. & Wei S. H. Structure-derived electronic and optical properties of transparent conducting oxides. Phys. Rev. B 71, 125129 (2005).

[b14] Shen X. *et al.* Phase transition of Zn_2_SnO_4_ nanowires under high pressure. J. Appl. Phys. 106, 113523 (2009).

[b15] Gracia L., Beltrán A. & Andrés J. A theoretical study on the pressure-induced phase transitions in the inverse spinel structure Zn_2_SnO_4_. J. Phys. Chem.C 115, 7740 (2011).

[b16] Zeng Y., Bing Y. F., Liu C., Zheng W. T. & Zou G. T. Hydrothermal synthesis and PL properties of Zn_2_SnO_4_ nanocubes. J. Lumin. 33, 716 (2012).

[b17] Guo Q. X. *et al.* Phase transformation in Sm_2_O_3_ at high pressure: *In situ* synchrotron X-ray diffraction study and ab initio DFT calculation. Solid State Communications 145, 5 (2008).

[b18] Zhang F. X. *et al.* Structural phase transitions of cubic Gd_2_O_3_ at high pressures. Physical Review B 78, 6 (2008).

[b19] Guo Q. X. *et al.* Cubic to tetragonal phase transformation in cold-compressed Pd nanocubes. Nano lett. 8, 972 (2008). 18237151 10.1021/nl0731217

[b20] Yang C. & Li S. Investigation of cohesive energy effects on size-dependent physical and chemical properties of nanocrystals. Phys. Rev. B 75, 165413 (2007).

[b21] Li Y. *et al.* Pressure effects on grain boundary, electrical and vibrational properties of the polycrystalline BaTeO_3_. Europhys. Lett. 98, 66006 (2012).

[b22] He C. Y. *et al.* *In situ* electrical impedance spectroscopy under high pressure on diamond anvil cell. Appl. Phys. Lett. 91, 092124 (2007).

[b23] Hamaker H. C. The london-van der waals attraction between spherical particles. Physica 4, 1058 (1937).

[b24] Mao H. K., Bell P. M., Shaner J. T. & Steinberg D. J. Specific volume measurements of Cu, Mo, Pd, and Ag and calibration of the ruby R_1_ fluorescence pressure gauge from 0.06 to 1 Mbar. J. Appl. Phys. 49, 3276 (1978).

[b25] Wang Y. *et al.* *In situ* impedance measurements in diamond anvil cell under high pressure. Rev. Sci. Instrum. 81, 013904 (2010). 20113110 10.1063/1.3282444

[b26] Han Y. H. *et al.* Integrated microcircuit on a diamond anvil for high-pressure electrical resistivity measurement. Appl. Phys. Lett. 86, 064104 (2005).

[b27] Gao C. X. *et al.* Accurate measurements of high pressure resistivity in a diamond anvil cell. Rev. Sci. Instrum. 76, 083912 (2005).

